# Salvage Therapy With Low-Dose Ruxolitinib Leads to a Significant Improvement in Bronchiolitis Obliterans Syndrome in Patients With cGVHD After Allogeneic Hematopoietic Stem Cell Transplantation

**DOI:** 10.3389/fphar.2021.668825

**Published:** 2021-06-28

**Authors:** Yanmin Zhao, Guifang OuYang, Jimin Shi, Yi Luo, Yamin Tan, Jian Yu, Huarui Fu, Xiaoyu Lai, Lizhen Liu, He Huang

**Affiliations:** ^1^ Bone Marrow Transplantation Center, The First Affiliated Hospital, School of Medicine, Zhejiang University, Hangzhou, China; ^2^ Institute of Hematology, Zhejiang University, Hangzhou, China; ^3^ Department of Hematology, Ningbo Hospital of Zhejiang University, Ningbo, China

**Keywords:** ruxolitinib, bronchiolitis obliterans syndrome, allogeneic hematopoietic stem cell transplantation, chronic graft versus host disease, pulmonary function

## Abstract

Bronchiolitis obliterans syndrome (BOS) is a life-threatening pulmonary manifestation of chronic graft versus host disease (cGVHD) post-allogeneic hematopoietic stem cell transplantation (HSCT), without clear standard of care. This study included 30 patients undergoing an allogeneic HSCT for a hematological malignancy and the outcomes with post-HSCT BOS treated with ruxolitinib as a salvage treatment were reviewed. After a median duration of ruxolitinib therapy of 9.25 (1.5–27) months, the best overall response (BOR) rate was 66.7%: three patients (10.0%) achieved complete remission, and 17 (56.7%) achieved partial remission. The median time from initiation of ruxolitinib to achieve the best responses was 3 months. Since initiating ruxolitinib, forced expiratory volume in 1 s of predicted (FEV1%pred) slightly increased after 3 and 6 months compared with measurements before ruxolitinib in responders. Only FEV1%pred mild decline before ruxolitinib with a ratio ≤15% was an independent predictor to achieve a response to ruxolitinib. Eleven patients (36.7%) had severe pulmonary infection of ≥3 grade. Following a median follow-up of 318 days after ruxolitinib, the 2-years incidence of nonrelapse mortality and 2-years overall survival rate after ruxolitinib among patients with BOS was 25.1 and 62.6%, respectively. Ruxolitinib is a promising treatment option to improve the prognosis of post-HSCT BOS.

## Introduction

Bronchiolitis obliterans syndrome (BOS) is a pulmonary fibroproliferative disorder characterized by irreversible narrowing and obliteration of the small airways. (Barker et al., 2014). In the setting of allogeneic hematopoietic stem cell transplantation (allo-HSCT), BOS is the most common pulmonary manifestation of moderate/severe chronic graft versus host disease (cGVHD), and the overall prevalence of BOS is up to 14% among patients who develop cGVHD (Au et al., 2011). BOS remains a leading cause of late morbidity and mortality as well as impaired quality of life after allo-HSCT. (Dudek et al., 2003; Williams et al., 2009; Chien et al., 2010; Barker et al., 2014).

Historically, systemic corticosteroids have formed the backbone of therapy for BOS, but their side effect profile makes them disadvantageous for prolonged use. Combination of inhaled corticosteroids and bronchodilators, azithromycin and montelukast early in BOS therapy could reduce total corticosteroids exposure (Bergeron et al., 2015; Kim et al., 2016; Williams et al., 2016). Other regimens include calcineurin inhibitors (CNIs); extracorporeal photopheresis (ECP) (Lucid et al., 2011; Del Fante et al., 2016; Hefazi et al., 2018); rituximab; (Brownback et al., 2017); etanercept; (Yanik et al., 2012); and tyrosine kinase inhibitors, mainly for their inhibitory effect on fibrosis via the platelet-derived growth factor pathway (Watanabe et al., 2015; Bergeron et al., 2017; Watanabe et al., 2017). Young patients with refractory severe BOS despite all interventions and best supportive care, may be evaluated for lung transplantation (Vogl et al., 2013; Greer et al., 2018). However, no consensus has been reached regarding the optimal therapy for BOS because of suboptimal responses to medical therapies, and effective therapeutic strategies are therefore needed to stabilize lung function, reduce corticosteroid dosing, and improve quality of life for patients with BOS after HSCT.

Ruxolitinib, a Janus-associated kinase (JAK) 1/2 inhibitor approved for the treatment of patients with intermediate-2 or high-risk primary myelofibrosis (Harrison et al., 2012), has shown excellent response rates in steroid-refractory GVHD (Zeiser et al., 2015; Khoury et al., 2018; Zhao et al., 2020a; Escamilla Gómez et al., 2020; Wu et al., 2021). Presently, studies on ruxolitinib administration in patients with BOS are still in a preliminary stage, with only two published case series, which indicated ruxolitinib is an effective steroid sparing agent in BOS due to cGVHD (Schoettler et al., 2019; Streiler et al., 2020). However, the effect of ruxolitinib on pulmonary function, its toxicity profile, and factors affecting its response have not been fully determined. All these concerns must be addressed urgently. Thus, this retrospective study aimed to investigate whether ruxolitinib therapy would lead to stabilization or even improvement in pulmonary function in patients with steroid-refractory BOS. Additionally, it sought to observe the infectious events during ruxolitinib therapy. It also determined the effect of ruxolitinib in corticosteroid dosing in these patients and the clinical factors affecting the response to ruxolitinib.

## Method

### Patients

All patients who underwent an allogeneic HSCT for a hematological malignancy between January 2014 and June 2019 at the First Affiliated Hospital of Zhejiang University School of Medicine and Ningbo Hospital of Zhejiang University were retrospectively screened. Data was retrieved from the transplant databases and electronic medical. The inclusion criteria were as follows: 1) patients aged ≥14 years; 2) diagnosed with BOS related to cGVHD according to the criteria of the National Institute of Health (NIH) consensus; 3) treated with systemic glucocorticosteroids (GCSs) in combination with therapy using inhaled corticosteroids fluticasone, azithromycin, and montelukast (FAM) with/without CNIs, and had worsening lung function, which was defined as a decline in forced expiratory volume in 1 s of predicted (FEV1%pred) from baseline by 5% or more; and 4) treated with ruxolitinib as a salvage treatment for at least 30 days. The exclusion criteria were as follows: 1) Responsive to other salvage therapy except ruxolitinib; 2) other pulmonary or infectious diseases, such as asthma, lung cancer, chronic obstructive pulmonary disease, pneumonia, or tuberculosis; and 3) a history of using other JAK inhibitors. All procedures were in accordance with the Declaration of Helsinki and approved by the ethics review committee of Zhejiang University. The author had full access to the data and took responsibility for its authenticity.

### BOS and cGVHD Diagnosis

BOS was diagnosed according to the National Institutes of Health 2014 criteria (Jagasia et al., 2015), namely 1) FEV1 <75% of that predicted or ≥10% absolute FEV1 decline over less than 2 years; 2) FEV1/forced vital capacity (FVC) < 70%; 3) absence of infection in the respiratory tract; and 4) one of the following two features of air trapping, that is, residual volume (RV) or predicted RV/total lung capacity >120% or evidence of air trapping on high-resolution computed tomography. cGVHD was defined according to the NIH Consensus Guidelines and classified as mild, moderate, or severe. (Jagasia et al., 2015).

### Pulmonary Function Test

Pulmonary function test (PFT) was routinely performed before HSCT and at any time when complaining of respiratory symptoms, such as dry cough, wheezing or dyspnea. After initiation of ruxolitinib, PFT was carried over time (0, 3, and 6 months) during the period of ruxolitinib therapy, using body plethysmography (MasterScreen PFT System, Jaeger Corp, Germany). If a patient had an episode of infection at the indicated time points, PFT was postponed to allow the resolution of infection.

### Ruxolitinib Therapy

Ruxolitinib was recommended at a dose of 5 mg orally, twice daily (BID), for patients weighing ≤60 kg, and 10 mg BID for patients weighing >60 kg. Adverse events were graded according to the Common Terminology Criteria for Adverse Events (CTCAE v4). If CTCAE grade 3 cytopenia occurred after ruxolitinib, the dose could be reduced according to the clinical situation. When ruxolitinib was started, the initial steroid was maintained for at least 4 weeks, after which a tapering schedule was commenced up at the discretion of the managing physician according to response.

### Therapeutic Response Definitions

The response to ruxolitinib was defined as complete response (CR) when clinical symptoms decreased, and FEV1%pred increased by >75%; partial response (PR) was considered when FEV1%pred levels increased or symptoms improved with the stabilization of FEV1%pred (among partial responders, good responders were those patients reaching FEV1%pred levels above 60%); and nonresponse (NR) consisted of progression disease (PD) and stable disease (SD). PD was considered when clinical and functional findings (mainly FEV1%pred decrease from baseline by 5% or more) worsened, while SD was considered when FEV1%pred decreased to less than 5% with stable symptoms.

### Statistical Analysis

Univariable analyses for treatment response risk factors were performed using the Fisher exact test and chi-square test. Logistic regression analysis was used to perform multivariable analysis for risk factors with *p* < 0.1 in univariate analysis. OS was defined as the time from stem cell infusion to death from any cause and calculated by Kaplan-Meier method. SPSS was used for the survival analysis (SPSS version 22.0.01; IBM, NY, United States). The proportional-hazards method was used to estimate the cumulative incidence of relapse and nonrelapse mortality (NRM). Relapse and nonrelapse mortality were competing risks for each other. The R statistical software (version 3.4.3; http://www.r-project.org) was used for the competing risk analysis.

## Results

### Patient Characteristics

**FIGURE 1:**
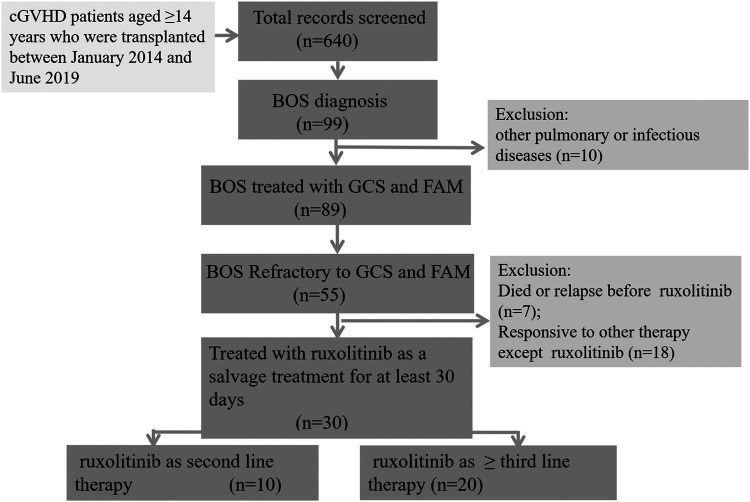
Flow diagram of the study cohort. Eighty-nine patients were diagnosed with BOS related to cGVHD, not related to other pulmonary or infectious disease. Among these 89 patients, 30 patients who were refractory disease to first-line glucocorticosteroids plus FAM and treated with ruxolitinib as ≥ second-line therapy, were retrospectively enrolled in this analysis. cGVHD, chronic graft versus host disease; BOS, bronchiolitis obliterans syndrome.

A total of 640 patients aged ≥14 years who were transplanted between January 2014 and June 2019 was screened. Eighty-nine patients were diagnosed with BOS related to cGVHD, not related to other pulmonary or infectious disease. Among these 89 patients, 30 patients who were refractory disease to first-line GCSs plus FAM and treated with ruxolitinib as second-line or ≥ third-line therapy, were retrospectively enrolled in this analysis (Figure 1). The patient characteristics are shown in Table 1. The median age was 27 (range 15–54) years at the time of transplantation. All the patients received a myeloablative conditioning regimen consisting of busulfan and cyclophosphamide. Antithymocyte globulin was also used in 22 (73.3%) patients, mainly for haploidentical stem cell transplantation. Fourteen (46.7%) patients received donor lymphocyte infusion as prophylaxis or preemptive intervention for relapse. Acute GVHD occurred in 21 patients, including 14 with I–II degree and 7 with III–IV degree disease. Chronic GVHD was present in all the 30 patients and graded as moderate in 6 (20%) and severe in 24 (80%) patients. For cGVHD, more than half of patients (56.7%) had three or more organs involved apart from the lungs. The prophylaxis for fungal disease included fluconazole (*n* = 11), voriconazole (*n* = 8), or posaconazole (*n* = 11).

**TABLE 1:** Characteristics of the study cohort at baseline.

Characteristic	N = 30
Gender, male, n (%)	13 (43.3%)
Recipient age at SCT (yrs), median (range)	27 (15–54)
Hematologic malignancy, n (%)	
AML/AMLL	16 (53.4%)
ALL	12 (40%)
MDS	1 (3.3%)
NK/T lymphoma	1 (3.3%)
Disease status at SCT, n (%)	
CR	27 (90%)
NR	3 (10%)
Donor type, n (%)	
matched unrelated	1 (3.3%)
matched sibling	8 (26.7%)
haplo-identical related donor	21 (70%)
Stem cell source, PBMSC, n (%)	30 (100%)
Conditioning regimens, MAC, n (%)	30 (100%)
ATG in conditioning regimens, n (%)	22 (73.3%)
Prophylaxis for GVHD	
CSA, MTX plus MMF	23 (76.7%)
FK506, MTX plus MMF	7 (23.3%)
Donor lymphocyte infusion, n (%)	14 (46.7%)
Acute GVHD grade, n (%)	
0	9 (30%)
I-II	14 (46.7%)
III-IV	7 (23.3%)
Chronic GVHD sites (except lung)	
Skin	20 (66.7%)
Oral	19 (63.3%)
eyes	14 (46.7%)
Liver	6 (20%)
Joints	6 (20%)
GI	5 (16.7%)
Extrathoracic cGVHD, n (%)	
1–2	13 (43.3%)
≥3	17 (56.7%)
Chronic GVHD grade, n (%)	
Moderate	6 (20%)
Severe	24 (80%)
Time to BOS from SCT, days, median (range)	452 (106–1902)
FEV1 at the time of HSCT, mean (range)	99.6 (81.2–125.1)
FEV1 before ruxolitinib, mean (range)	43.8 (25.8–71.2)

Before SCT, all the patients had normal PFTs, with FEV1%pred ranging from 81.2 to 125.1 (mean, 99.6). The median time from transplantation to the onset of BOS was 453 (range, 106–1902) days. At the time of BOS diagnosis, most patients presented with moderate or severe obstruction (mean FEV1%pred: 55.5%, range 39.2–76.0%). All the patients received systemic prednisone after the diagnosis of BOS, with a median dose of 0.48 (range, 0.25–0.9) mg/(kg ⋅ day), along with FAM therapy and/or CNIs, as a first-line treatment regimen. Therapies beyond front-line GCS, FAM, and CNIs included MMF (*n* = 14), imatinib (*n* = 12), MSCs infusion (*n* = 5), rituximab (*n* = 2), recombinant human α receptor antibody fusion protein of tumor necrosis factor (rhRPTN, etanercept) (*n* = 1), and pirfenidone (*n* = 1). Despite the aforementioned therapeutic intervention, these 30 patients had worsening lung function (decreased FEV1%pred from baseline by 5% or more), with or without worsening clinical symptoms; therefore, they switched to ruxolitinib.

### Therapeutic Response to Ruxolitinib

**FIGURE 2:**
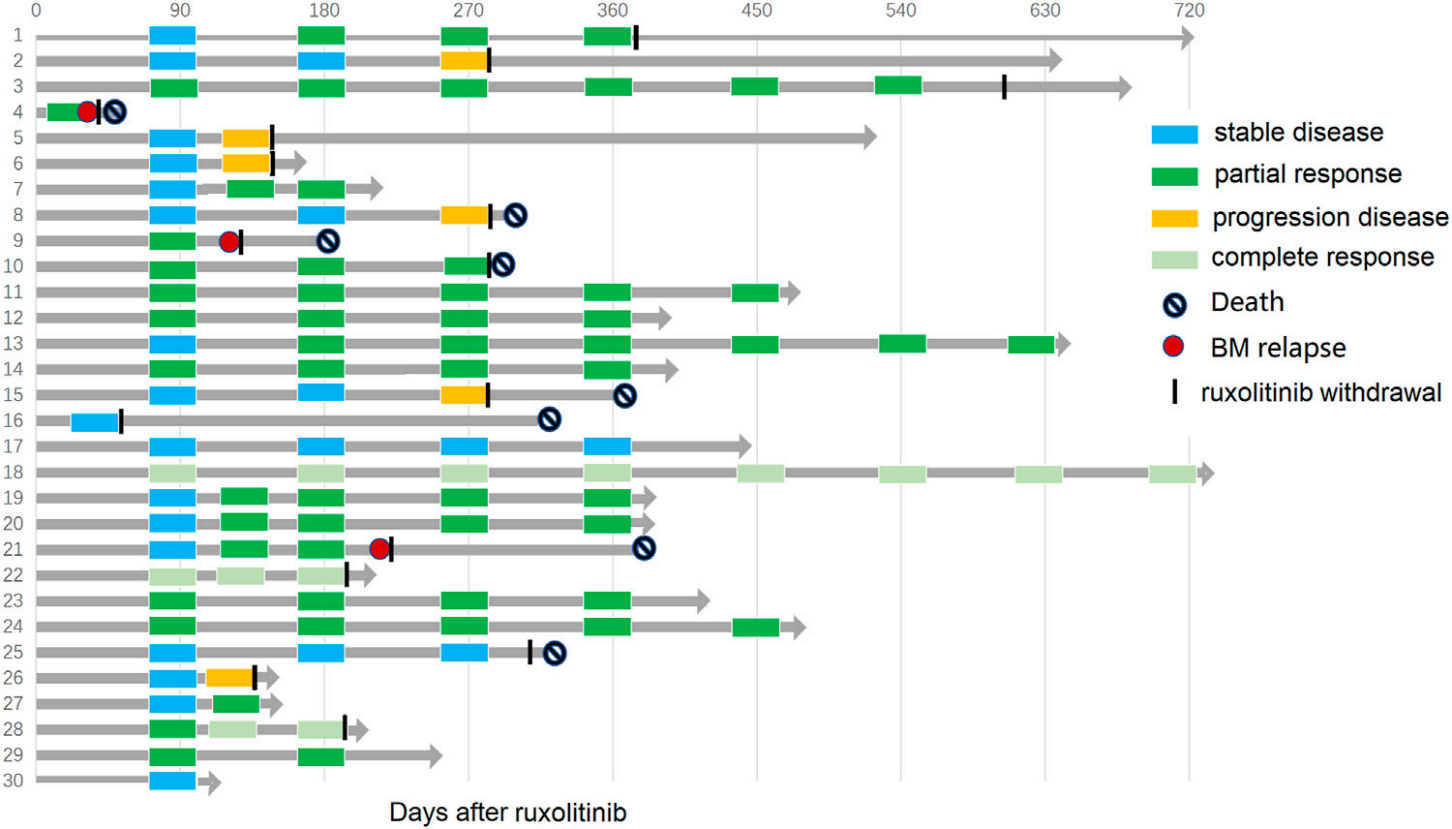
Swimmer plot depicting response, duration of response, and patient outcome with ruxolitinib for all 30 patients.

Ruxolitinib was given after a median time of 125 (range 27–1,598) days from BOS diagnosis, as the second and ≥ third line of therapy in 33.3% (10/30) and 66.7% (20/30) of patients, respectively. After a median duration of ruxolitinib therapy of 9.25 (1.5–27) months, the best overall response rate (BOR) was 66.7%: three patients (10.0%) achieved a CR, and 17 (56.7%) achieved a PR. The ORR at 180 days was 56.7%. The median time from initiation of ruxolitinib to achieving the best responses was 3 (range, 1.5–8) months. Ten patients (33.3%) were nonresponders, including four with SD and six with progression. At the last follow-up, ruxolitinib was stopped in 16 patients due to continuous good response (FEV1%pred >60%, *n* = 2; FEV1%pred >75%, *n* = 2), bone marrow relapse of primary malignancy (*n* = 3), severe infectious complications (*n* = 3), and BOS progression (*n* = 6) (Figure 2).

**FIGURE 3:**
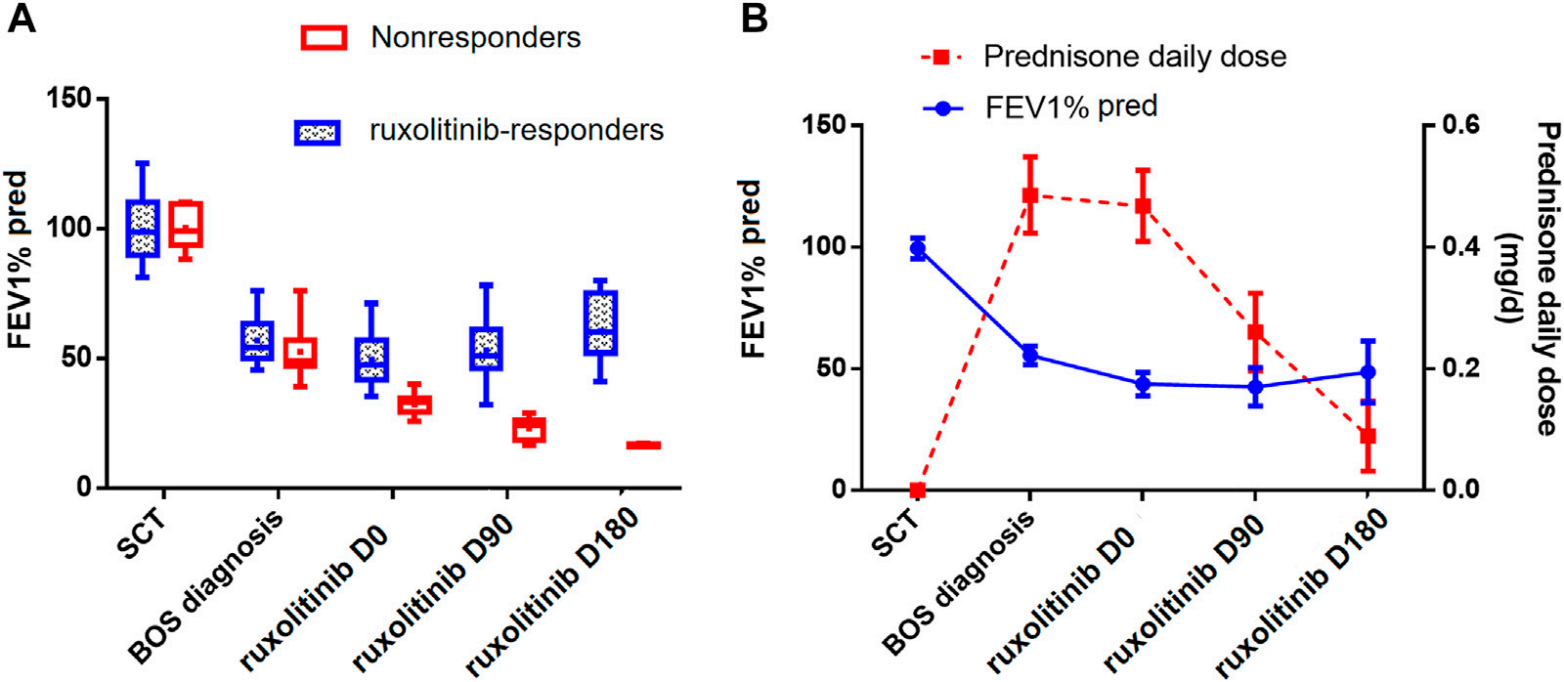
**(A)** Distribution of FEV1%pred over time in ruxolitinib responders and nonresponders. **(B)** Distribution of FEV1% pred and prednisone daily dose over time in all ruxolitinib-treated patients. All the parameters were represented by the mean values and 95%CI. FEV1%pred, forced expiratory volume in 1 s of predicted; BOS, bronchiolitis obliterans syndrome; SCT, stem cell transplantation.

Before the initiation of ruxolitinib, the mean FEV1%pred was 43.8 (range, 25.8–71.2). Since initiating ruxolitinib, PFTs improved over time in the 20 responders, whose FEV1%pred increased after 3 and 6 months compared with measurements before ruxolitinib (mean FEV1%pred in month 0 vs. month 3 vs. month 6: 49.5 vs. 53.0 vs. 60.5; month 0 vs. month 6: *p* = 0.029) (Figure 3A). An average reduction in prednisone dose of 44.3% after 3 months of ruxolitinib therapy and 80.9% after 6 months of ruxolitinib therapy was reported (Figure 3B). After a median follow-up of 318 (range, 53–730) days after ruxolitinib, nine responders (9/20, 45%) were able to stop steroids and 11 were able to wean steroids to 0.2 mg/(kg ⋅ day) with stable symptoms.

Patients with FEV1%pred >39% when receiving ruxolitinib were more likely to achieve a response compared with those with FEV1%pred ≤39%. (ORR 89.5 vs. 27.3%, *p* = 0.001). Also, the decline ratio of FEV1%pred from BOS diagnosis to ruxolitinib initiation ranged from 6.6 to 56.6%, with a median value of 15.0%. The mild-decline group with the FEV1%pred decline ratio ≤15% showed a significantly higher response rate compared with the severe-decline group whose FEV1%pred decline ratio was more than 15% (ORR 100 vs. 28.6%, *p* < 0.001). Other features related to a better response might be the use of ruxolitinib as the second line of therapy, compared with ≥third line (ORR 90.9 vs. 52.6%, *p* = 0.037). No difference in ORR was observed between early ruxolitinib group (interval from BOS diagnosis to ruxolitinib <6 months) and late ruxolitinib group (interval from BOS diagnosis to ruxolitinib ≥6 months) (61.1 vs. 75%, *p* = 0.350). Also, no significant differences related to response in age, sex, donor type, DLI history, chronic GVHD sites, BOS onset time and steroid dose at start of ruxolitinib were found. Variables with *p* < 0.05 in the univariate analysis were included in the later multivariate logistic regression analysis: FEV1%pred at ruxolitinib initiation; FEV1%pred decline ratio from BOS diagnosis to ruxolitinib initiation; ruxolitinib as different lines of salvage treatment. Only FEV1%pred mild decline before ruxolitinib was an independent predictor to achieve a response to ruxolitinib. (HR = 0.096, 95% CI 0.026–0.353, *p* = 0.001) (Table 2).

**TABLE 2:** Clinical univariate and multivariate analysis for achieving a response to ruxolitinib.

Variable	Univariate		Multivariate	
	ORR (%)	P	Hazard ratio (CI 95%)	P
Ruxolitinib salvage therapy		0.037		
2nd line	90.9			
≥3rd line	52.6			
FEV1 at ruxolitinib initiation				
≤39%	27.3	0.001		
>39%	89.5			
FEV1 decline ratio from BOS diagnosis to ruxolitinib initiation				
≤15%	100	0.001	0.096 (95% CI 0.026–0.35)	0.001
>15%	28.6		1	

## Adverse Events

Any grade hematological toxicities were observed in 4 (13.3%) patients, and for grade ≥3 cytopenia, only one patient had grade ≥3 anemia. Regarding the nonhematological toxicities, two patients developed grade 3 hypertriglyceridemia. Infection-related events were frequent in the present cohort. Of the 30 patients, 100% were cytomegalovirus (CMV) IgG positive, with a CMV-seropositive donor. CMV reactivation indicated as CMV DNAemia was observed in three patients (10%). Epstein–Barr virus (EBV) reactivation was observed in 11 patients (36.7%). None of the patients developed CMV disease or EBV post-transplant lymphoproliferative disorder. Hepatitis B virus was re-activated in two patients (6.7%), both of whom were hepatitis B surface antigen-negative (HBsAg-)/hepatitis B core antibody-positive (HBcAb+)/hepatitis B surface antibody negative (HBsAb-) without any hepatitis B prophylaxis. Fourteen patients (46.7%) were complicated with pulmonary infection ≥2 grade, including invasive pulmonary fungal diseases in 10 patients, pulmonary bacterial in three patients, and mixed infection (nocardiosis/Aspergillus fumigatus/*Klebsiella pneumoniae*) in one patient. The risk for pulmonary fungal infection was 54.5% in fluconazole prophylaxis group and 26.3% in voriconazole or posaconazole group (*p* = 0.238). The incidence of pulmonary infection was higher in the nonresponders than in the responders (89.0 vs. 46.7%, *p* = 0.019). No bacteremia occurred in this cohort.

## Relapse and Survival Among Patients With BOS Treated With Ruxolitinib

**FIGURE 4:**
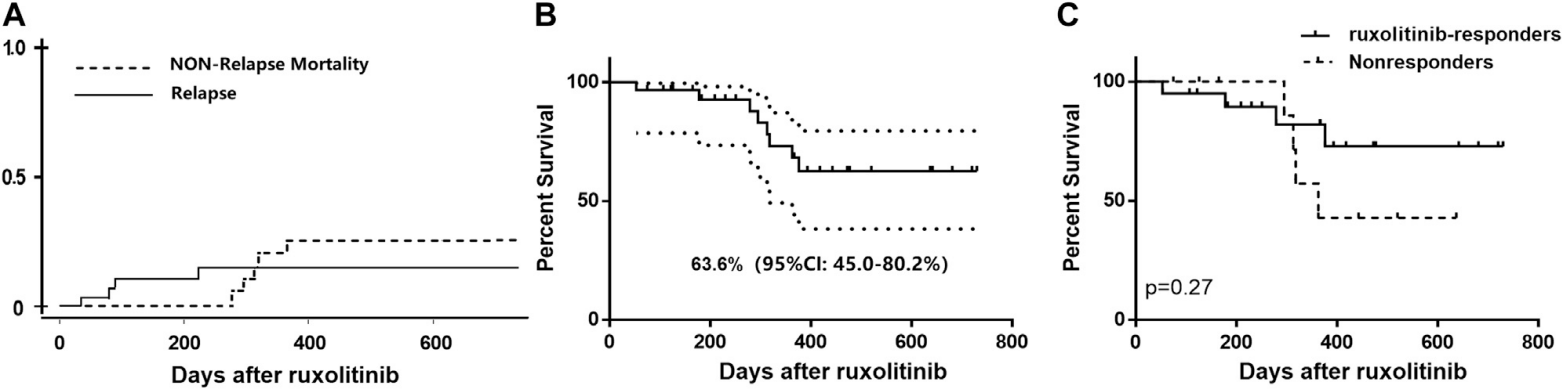
**(A)** Cumulative incidence of relapse and nonrelapse mortality of all the patients with BOS receiving ruxolitinib; **(B)** Overall survival curve of all ruxolitinib-treated patients; **(C)** Overall survival of ruxolitinib-responders and nonresponders.

After a median follow-up of 318 (range, 53–730) days after ruxolitinib initiation, 4 out of 30 patients experienced a relapse of primary malignancy, including three bone marrow relapse and one with isolated extramedullary relapse. The cumulative 1-year incidence of relapse of the underlying malignancy was 14.7% (95%CI: 4.4–30.9%) after ruxolitinib. Eventually, eight patients died (26.7%), with causes of death predominantly resulting from infection (severe pulmonary infection/respiratory failure, *n* = 4; severe hepatitis B/hepatorenal syndrome, *n* = 1) and bone marrow relapse of original malignant disease (*n* = 3). The 2-years incidence of NRM and 2-years OS rate after ruxolitinib among patients with BOS were 25.1% (95%CI: 8.8–45.5%) and 62.6% (95%CI: 45.0–80.2%), respectively. There is no statistically significant difference in survival rate in ruxolitinib-responders and no responding patients (1-year OS rate: 72.9 vs. 42.9%, *p* = 0.27) (Figure 4).

## Discussion

Ruxolitinib, a potent anti-GVHD drug, might be a promising therapeutic option for BOS after HSCT. The present study added to a growing body of evidence for the use of ruxolitinib in SR-BOS related to cGVHD. In these heavily pretreated patients with BOS, ruxolitinib induced a relatively high response (ORR 66.7%), including FEV1 improvement and steroid sparing. The 2-years OS rate after ruxolitinib among patients with BOS was 62.6%. The present study suggests such heavily pretreated patients might benefit from salvage therapy with ruxolitinib. More importantly, relapse of the underlying malignancy in ruxolitinib-exposed patients was low (14.7%). This frequency is comparable to other currently applied immunosuppressive drugs, and other data from murine models proved that ruxolitinib does not impair the graft-vs.-leukemia (GvL) activity of alloreactive donor T cells, (Choi et al., 2014), indicating that ruxolitinib treatment is not linked to a higher relapse risk.

The pathophysiology of BOS following allo-HSCT is still not completely understood. Current evidence suggests that regulatory T (Treg) cells are effective in controlling chronic GVHD and BOS, as this cell subset can inhibit alloreactive T-cell and germinal center (GC) B-cell reactions to produce pathogenic antibodies for deposition in BOS lesion sites. (McDonald-Hyman et al., 2016). Due to the key role of JAK1/2-STAT3 pathways on T cells activation, JAK 1/2 inhibitor ruxolitinib may reduce donor alloreactive T-cell expansion and inflammatory cytokine production, and meanwhile preserve or even promote Treg recovery post-transplantation through sparing JAK3-STAT5 pathway (Spoerl et al., 2014), explaining the mechanism of action of ruxolitinib to ameliorate cGVHD related BOS. Furthermore, some components such as several chemokines, and other profibrotic factors accumulate in the airway, facilitate macrophage infiltration and collagen deposition, and hence lead to airway remodeling, and eventually, airway obstruction (Liu et al., 2001; Barker et al., 2014). In GVHD models, ruxolitinib was shown to decrease the production of chemokines and profibrotic cytokine (Spoerl et al., 2014), and in the neutrophilic asthma mouse model, it decreased the IL-17A level in bronchoalveolar lavage fluid (BALF), reduced the proportion of Th17 cells in lung tissue, and finally reduced airway inflammation. As for macrophages, ruxolitinib was shown to inhibit macrophage infiltration and prevent the upregulation of various proinflammatory cytokines in human macrophages (Maschalidi et al., 2016; Febvre-James et al., 2018). Collectively, the potent immunosuppressive and anti-inflammatory activities of ruxolitinib might be valuable for the management of BOS in the allogeneic stem cell transplantation setting.

This study demonstrated that while many patients showed an improvement in PFT with ruxolitinib, a substantial proportion of patients failed to respond to this treatment. Identifying potential responders may be more important than implementing ruxolitinib across the board in patients with BOS. Theoretically, early “inflammatory” phase might be more prone to respond to ruxolitinib than late “fibrotic” phase due to the potent anti-inflammatory effect of the drug. Actually, in our retrospective study, there is no significant difference in ORR between early ruxolitinib group and delayed ruxolitinib group. Only one element was identified to be independently related to achieving responses: FEV1%pred mild-decline before ruxolitinib with the FEV1%pred decline ratio ≤15%. It was consistent with a previous study by Kwok et al., who found that patients with BOS who manifested initial rapid lung function decline within 3 months of BOS diagnosis had significantly poorer lung function and worse OS compared with those with a gradual decline (Kwok et al., 2019). Unfortunately, despite interventions, a rapid persistent decline in FEV1 was associated with poor prognosis (Ahn et al., 2015). The ability of ruxolitinib to reverse the natural history of BOS in the FEV1 rapid decline cohort was not observed. Considering that the majority of early ruxolitinib group who moved quickly to ruxolitinib therapy belonged to the BOS progressive population, with a severe deteriorating pulmonary function, thereby the advantage of early inflammatory nature might be obscured by the more important element: rapid lung function decline. And the causes of the rapid deterioration of lung function need to be clarified and other treatment strategies need to be explored.

Ruxolitinib might increase the incidence of infections, which remained a major concern. Increasing evidence suggests that ruxolitinib may impair both innate and adaptive immunity. In detail, ruxolitinib was shown to reduced and functionally impaired dendritic cell (DC) leading to impaired T-cell activation (Heine et al., 2013a). Inhibition of JAK1 impairs cytokine production, which may result in reduced control of silent infections and increased risk of reactivation of latent infections (Heine et al., 2013b) In particular, ruxolitinib drastically reduces the quantity of natural killer (NK) cells (Schönberg et al., 2015). All these effects may result in varying degrees of immunodeficiency, leading to high risks for infectious complications. Besides, it's worth noting that the incidence of pulmonary infection (46.7%) in patients with BOS in the present study was obviously higher than that in patients with myeloproliferative neoplasms receiving ruxolitinib form Comfort II/JUMP (5.3–13.1%) and that in another cohort receiving ruxolitinib as GVHD prophylaxis (Lussana et al., 2018; Zhao et al., 2020b). indicating this high rate of coinfections was not only attributed to the effects of JAK1/2 inhibition on immunosurveillance but also associated with the innate characteristics of BOS. Like patients with bronchiectasis from other causes, patients with BOS can be colonized with Gram-negative organisms and fungus (mostly *Aspergillu*s) (Hayes et al., 2013). Interestingly, in the present study, the incidence of pulmonary infection was higher in the nonresponders than in the responders. For nonresponders who relied on long-term high-dose steroids, ruxolitinib might worsen the infection status, inciting chemokines, accelerating BOS progression, and finally forming a vicious circle (Shino et al., 2013). Also, in our study, patients receiving fluconazole as antifungal prophylaxis might be even more at risk from fungal infection, indicating strong consideration should be given to voriconazole or posaconazole for prophylaxis, as mentioned by Williams, K.M (Williams, 2017). Taken together, the present study highlighted the importance of the assessment of risk factors for infectious events, along with anti-infective prophylaxis prior to initiation of ruxolitinib in patients who were already immunocompromised.

As it is considered standard of care, that every patient is required to monitor PFTs after allogeneic SCT, and in the long time, this implementation is highly recommended, thus the main limitation of the study is insufficient monitoring of PFTs in asymptomatic patients due to inadequate medical resources. The second imitation is that a small number of subjects and a retrospective nonrandomized design of this study might make a bias. However, our data still indicated ruxolitinib as a promising treatment option to improve the prognosis of post-HSCT BOS, with pulmonary function stabilization or even improvement and steroid sparing. This approach might provide a reference for the treatment of BOS in other causes including autoimmune and post lung transplantation. Also, the substantial clinical benefits and efficacy of ruxolitinib on BOS need to be balanced against infection risks. Further, hopefully prospective randomized controlled trials, are needed to definitely prove the efficacy of ruxolitinib in treatment of cGVHD-related BOS.

## Data Availability

The original contributions presented in the study are included in the article/supplementary material, further inquiries can be directed to the corresponding author.

## References

[B1] Ahn J. H. Jo K.-W. Song J. W. Shim T. S. Lee S. W. Lee J. S. (2015). Prognostic Role of FEV1for Survival in Bronchiolitis Obliterans Syndrome after Allogeneic Hematopoietic Stem Cell Transplantation. Clin. Transpl. 29 (12), 1133–1139. 10.1111/ctr.12638 26383085

[B2] Au B. K. C. Au M. A. Chien J. W. (2011). Bronchiolitis Obliterans Syndrome Epidemiology after Allogeneic Hematopoietic Cell Transplantation. Biol. Blood Marrow Transplant. 17 (7), 1072–1078. 10.1016/j.bbmt.2010.11.018 21126596 PMC3061253

[B3] Barker A. F. Bergeron A. Rom W. N. Hertz M. I. (2014). Obliterative Bronchiolitis. N. Engl. J. Med. 370 (19), 1820–1828. e-pub ahead of print 2014/05/09. 10.1056/NEJMra1204664 24806161

[B4] Bergeron A. Chevret S. Chagnon K. Godet C. Bergot E. Peffault de Latour R. (2015). Budesonide/Formoterol for Bronchiolitis Obliterans after Hematopoietic Stem Cell Transplantation. Am. J. Respir. Crit. Care Med. 191 (11), 1242–1249. 10.1164/rccm.201410-1818OC 25835160

[B5] Bergeron A. Chevret S. Granata A. Chevallier P. Vincent L. Huynh A. (2017). Effect of Azithromycin on Airflow Decline-free Survival after Allogeneic Hematopoietic Stem Cell Transplant. JAMA 318 (6), 557–566. 10.1001/jama.2017.9938 28787506 PMC5817485

[B6] Brownback K. R. Thomas L. A. McGuirk J. P. Ganguly S. Streiler C. Abhyankar S. (2017). Effect of Rituximab on Pulmonary Function in Bronchiolitis Obliterans Syndrome Due to Graft-Versus-Host-Disease. Lung 195 (6), 781–788. e-pub ahead of print 2017/09/13. 10.1007/s00408-017-0051-0 28894914

[B7] Chien J. W. Duncan S. Williams K. M. Pavletic S. Z. (2010). Bronchiolitis Obliterans Syndrome after Allogeneic Hematopoietic Stem Cell Transplantation-An Increasingly Recognized Manifestation of Chronic Graft-Versus-Host Disease. Biol. Blood Marrow Transplant. 16 (1 Suppl. l), S106–S114. 10.1016/j.bbmt.2009.11.002 19896545 PMC3189470

[B8] Choi J. Cooper M. L. Alahmari B. Ritchey J. Collins L. Holt M. (2014). Pharmacologic Blockade of JAK1/JAK2 Reduces GvHD and Preserves the Graft-Versus-Leukemia Effect. PLoS One 9 (10), e109799. 10.1371/journal.pone.0109799 25289677 PMC4188578

[B9] Del Fante C. Galasso T. Bernasconi P. Scudeller L. Ripamonti F. Perotti C. (2016). Extracorporeal Photopheresis as a New Supportive Therapy for Bronchiolitis Obliterans Syndrome after Allogeneic Stem Cell Transplantation. Bone Marrow Transpl. 51 (5), 728–731. 10.1038/bmt.2015.324 26726939

[B10] Dudek A. Z. Mahaseth H. DeFor T. E. Weisdorf D. J. (2003). Bronchiolitis Obliterans in Chronic Graft-Versus-Host Disease: Analysis of Risk Factors and Treatment Outcomes. Biol. Blood Marrow Transplant. 9 (10), 657–666. 10.1016/s1083-8791(03)00242-8 14569562

[B11] Escamilla Gómez V. García-Gutiérrez V. García-Gutiérrez V. López Corral L. García Cadenas I. Pérez Martínez A. (2020). Ruxolitinib in Refractory Acute and Chronic Graft-Versus-Host Disease: a Multicenter Survey Study. Bone Marrow Transpl. 55 (3), 641–648. e-pub ahead of print 2019/11/09. 10.1038/s41409-019-0731-x PMC7051903 31700138

[B12] Febvre-James M. Lecureur V. Augagneur Y. Mayati A. Fardel O. (2018). Repression of Interferon β-regulated Cytokines by the JAK1/2 Inhibitor Ruxolitinib in Inflammatory Human Macrophages. Int. immunopharmacol. 54, 354–365. e-pub ahead of print 2017/12/05. 10.1016/j.intimp.2017.11.032 29202299

[B13] Greer M. Berastegui C. Jaksch P. Benden C. Aubert J. Roux A. (2018). Lung Transplantation after Allogeneic Stem Cell Transplantation: a Pan-European Experience. Eur. Respir. J. 51 (2), 1701330. e-pub ahead of print 2018/02/16. 10.1183/13993003.01330-2017 29444913

[B14] Harrison C. Kiladjian J.-J. Al-Ali H. K. Gisslinger H. Waltzman R. Stalbovskaya V. (2012). JAK Inhibition with Ruxolitinib versus Best Available Therapy for Myelofibrosis. N. Engl. J. Med. 366 (9), 787–798. 10.1056/NEJMoa1110556 22375970

[B15] Hayes D. Jr. Weiland A. Kirkby S. Galantowicz M. McConnell P. I. Tobias J. D. (2013). Gram-negative Infection and Bronchiectasis in Lung Transplant Recipients with Bronchiolitis Obliterans Syndrome. Thorac. Cardiovasc. Surg. 61 (3), 240–245. 10.1055/s-0032-1322619 23225511

[B16] Hefazi M. Langer K. J. Khera N. Adamski J. Roy V. Winters J. L. (2018). Extracorporeal Photopheresis Improves Survival in Hematopoietic Cell Transplant Patients with Bronchiolitis Obliterans Syndrome without Significantly Impacting Measured Pulmonary Functions. Biol. Blood Marrow Transplant. 24 (9), 1906–1913. 10.1016/j.bbmt.2018.04.012 29679771

[B17] Heine A. Held S. A. E. Daecke S. N. Wallner S. Yajnanarayana S. P. Kurts C. (2013). The JAK-Inhibitor Ruxolitinib Impairs Dendritic Cell Function *In Vitro* and *In Vivo* . Blood 122 (7), 1192–1202. 10.1182/blood-2013-03-484642 23770777

[B18] Heine A. Brossart P. Wolf D. (2013). Ruxolitinib Is a Potent Immunosuppressive Compound: Is it Time for Anti-infective Prophylaxis?. Blood 122 (23), 3843–3844. 10.1182/blood-2013-10-531103 24288410

[B19] Jagasia M. H. Greinix H. T. Arora M. Williams K. M. Wolff D. Cowen E. W. (2015). National Institutes of Health Consensus Development Project on Criteria for Clinical Trials in Chronic Graft-Versus-Host Disease: I. The 2014 Diagnosis and Staging Working Group Report. Biol. Blood Marrow Transplant. 21 (3), 389–401. 10.1016/j.bbmt.2014.12.001 25529383 PMC4329079

[B20] Khoury H. J. Langston A. A. Kota V. K. Wilkinson J. A. Pusic I. Jillella A. (2018). Ruxolitinib: a Steroid Sparing Agent in Chronic Graft-Versus-Host Disease. Bone Marrow Transpl. 53 (7), 826–831. 10.1038/s41409-017-0081-5 PMC6041160 29367708

[B21] Kim S. W. Rhee C. K. Kim Y. J. Lee S. Kim H. J. Lee J. W. (2016). Therapeutic Effect of Budesonide/formoterol, Montelukast and N-Acetylcysteine for Bronchiolitis Obliterans Syndrome after Hematopoietic Stem Cell Transplantation. Respir. Res. 17 (1), 63. 10.1186/s12931-016-0380-1 27229850 PMC4882858

[B22] Kwok W. C. Liang B. M. Lui M. M. S. Tam T. C. C. Sim J. P. Y. Tse E. W. C. (2019). Rapid versus Gradual Lung Function Decline in Bronchiolitis Obliterans Syndrome after Haematopoietic Stem Cell Transplantation Is Associated with Survival Outcome. Respirology 24 (5), 459–466. 10.1111/resp.13472 30663178

[B23] Liu J.-Y. Sime P. J. Wu T. Warshamana G. S. Pociask D. Tsai S.-Y. (2001). Transforming Growth Factor- β1Overexpression in Tumor Necrosis Factor- α Receptor Knockout Mice Induces Fibroproliferative Lung Disease. Am. J. Respir. Cel Mol Biol 25 (1), 3–7. 10.1165/ajrcmb.25.1.4481 11472967

[B24] Lucid C. E. Savani B. N. Engelhardt B. G. Shah P. Clifton C. Greenhut S. L. (2011). Extracorporeal Photopheresis in Patients with Refractory Bronchiolitis Obliterans Developing after Allo-SCT. Bone Marrow Transpl. 46 (3), 426–429. 10.1038/bmt.2010.152 20581885

[B25] Lussana F. Cattaneo M. Rambaldi A. Squizzato A. (2018). Ruxolitinib-associated Infections: A Systematic Review and Meta-Analysis. Am. J. Hematol. 93 (3), 339–347. e-pub ahead of print 2017/11/19. 10.1002/ajh.24976 29150886

[B26] Maschalidi S. Sepulveda F. E. Garrigue A. Fischer A. de Saint Basile G. (2016). Therapeutic Effect of JAK1/2 Blockade on the Manifestations of Hemophagocytic Lymphohistiocytosis in Mice. Blood 128 (1), 60–71. 10.1182/blood-2016-02-700013 27222478

[B27] McDonald-Hyman C. Flynn R. Panoskaltsis-Mortari A. Peterson N. MacDonald K. P. A. Hill G. R. (2016). Therapeutic Regulatory T-Cell Adoptive Transfer Ameliorates Established Murine Chronic GVHD in a CXCR5-dependent Manner. Blood 128 (7), 1013–1017. 10.1182/blood-2016-05-715896 27385791 PMC4990850

[B28] Schoettler M. Duncan C. Lehmann L. Furutani E. Subramaniam M. Margossian S. (2019). Ruxolitinib Is an Effective Steroid Sparing Agent in Children with Steroid Refractory/dependent Bronchiolitis Obliterans Syndrome after Allogenic Hematopoietic Cell Transplantation. Bone Marrow Transpl. 54 (7), 1158–1160. 10.1038/s41409-019-0450-3 PMC7988479 30683905

[B29] Schönberg K. Rudolph J. Vonnahme M. Parampalli Yajnanarayana S. Cornez I. Hejazi M. (2015). JAK Inhibition Impairs NK Cell Function in Myeloproliferative Neoplasms. Cancer Res. 75 (11), 2187–2199. 10.1158/0008-5472.CAN-14-3198 25832652

[B30] Shino M. Y. Weigt S. S. Li N. Palchevskiy V. Derhovanessian A. Saggar R. (2013). CXCR3 Ligands Are Associated with the Continuum of Diffuse Alveolar Damage to Chronic Lung Allograft Dysfunction. Am. J. Respir. Crit. Care Med. 188 (9), 1117–1125. 10.1164/rccm.201305-0861OC 24063316 PMC3863740

[B31] Spoerl S. Mathew N. R. Bscheider M. Schmitt-Graeff A. Chen S. Mueller T. (2014). Activity of Therapeutic JAK 1/2 Blockade in Graft-Versus-Host Disease. Blood 123 (24), 3832–3842. 10.1182/blood-2013-12-543736 24711661

[B32] Streiler C. Shaikh F. Davis C. Abhyankar S. Brownback K. R. (2020). Ruxolitinib Is an Effective Steroid Sparing Agent in Bronchiolitis Obliterans Due to Chronic Graft-Versus-Host-Disease. Bone Marrow Transpl. 55 (6), 1194–1196. e-pub ahead of print 2019/09/06. 10.1038/s41409-019-0662-6 31484991

[B33] Vogl U. M. Nagayama K. Bojic M. Hoda M. A. R. Klepetko W. Jaksch P. (2013). Lung Transplantation for Bronchiolitis Obliterans after Allogeneic Hematopoietic Stem Cell Transplantation. Transplantation 95 (4), 623–628. 10.1097/TP.0b013e318277e29e 23274967

[B34] Watanabe S. Waseda Y. Kimura H. Takato H. Ohata K. Kondo Y. (2015). Imatinib for Bronchiolitis Obliterans after Allogeneic Hematopoietic Stem Cell Transplantation. Bone Marrow Transpl. 50 (9), 1250–1252. 10.1038/bmt.2015.120 26052911

[B35] Watanabe S. Kasahara K. Waseda Y. Takato H. Nishikawa S. Yoneda T. (2017). Imatinib Ameliorates Bronchiolitis Obliterans via Inhibition of Fibrocyte Migration and Differentiation. J. Heart Lung Transplant. 36 (2), 138–147. 10.1016/j.healun.2016.06.001 27388852

[B36] Williams K. M. Chien J. W. Gladwin M. T. Pavletic S. Z. (2009). Bronchiolitis Obliterans after Allogeneic Hematopoietic Stem Cell Transplantation. JAMA 302 (3), 306–314. 10.1001/jama.2009.1018 19602690 PMC7357209

[B37] Williams K. M. Cheng G.-S. Pusic I. Jagasia M. Burns L. Ho V. T. (2016). Fluticasone, Azithromycin, and Montelukast Treatment for New-Onset Bronchiolitis Obliterans Syndrome after Hematopoietic Cell Transplantation. Biol. Blood Marrow Transplant. 22 (4), 710–716. e-pub ahead of print 2015/10/18. 10.1016/j.bbmt.2015.10.009 26475726 PMC4801753

[B38] Williams K. M. (2017). How I Treat Bronchiolitis Obliterans Syndrome after Hematopoietic Stem Cell Transplantation. Blood 129 (4), 448–455. 10.1182/blood-2016-08-693507 27856461 PMC5270387

[B39] Wu H. Shi J. Luo Y. Tan Y. Zhang M. Lai X. (2021). Evaluation of Ruxolitinib for Steroid-Refractory Chronic Graft-Vs-Host Disease after Allogeneic Hematopoietic Stem Cell Transplantation. JAMA Netw. Open 4 (1), e2034750. 10.1001/jamanetworkopen.2020.34750 33502484 PMC7841467

[B40] Yanik G. A. Mineishi S. Levine J. E. Kitko C. L. White E. S. Vander Lugt M. T. (2012). Soluble Tumor Necrosis Factor Receptor: Enbrel (Etanercept) for Subacute Pulmonary Dysfunction Following Allogeneic Stem Cell Transplantation. Biol. Blood Marrow Transplant. 18 (7), 1044–1054. 10.1016/j.bbmt.2011.11.031 22155140 PMC4462521

[B41] Zeiser R. Burchert A. Lengerke C. Verbeek M. Maas-Bauer K. Metzelder S. K. (2015). Ruxolitinib in Corticosteroid-Refractory Graft-Versus-Host Disease after Allogeneic Stem Cell Transplantation: a Multicenter Survey. Leukemia 29 (10), 2062–2068. e-pub ahead of print 2015/08/01. 10.1038/leu.2015.212 26228813 PMC4854652

[B42] Zhao Y. Wu H. Shi J. Luo Y. Li X. Lan J. (2020). Ruxolitinib Combined with Etanercept Induce a Rapid Response to Corticosteroid‐refractory Severe Acute Graft vs Host Disease after Allogeneic Stem Cell Transplantation: Results of a Multi‐center Prospective Study. Am. J. Hematol. 95 (9), 1075–1084. 10.1002/ajh.25898 32510625

[B43] Zhao Y. Shi J. Luo Y. Gao F. Tan Y. Lai X. (2020). Calcineurin Inhibitors Replacement by Ruxolitinib as Graft-Versus-Host Disease Prophylaxis for Patients after Allogeneic Stem Cell Transplantation. Biol. Blood Marrow Transplant. 26 (5), e128–e133. 10.1016/j.bbmt.2020.01.012 31982545

